# Spanish language opportunities in genetic counseling training programs in the United States

**DOI:** 10.1002/jgc4.1958

**Published:** 2024-08-27

**Authors:** Maria Katia Vine, Elizabeth Kellom, Ashley Kuhl, Laurie Simone, Charité N. Ricker, Laura Birkeland

**Affiliations:** ^1^ School of Medicine and Public Health University of Wisconsin Madison Wisconsin USA; ^2^ Waisman Center Madison Wisconsin USA; ^3^ Hackensack University Medical Center Hackensack New Jersey USA; ^4^ Keck School of Medicine University of Southern California Los Angeles California USA

**Keywords:** diversity, education, genetic counseling, language concordance, Spanish

## Abstract

This cross‐sectional survey study explores ongoing initiatives to foster diversity and inclusivity within the field of genetic counseling, specifically focusing on opportunities within graduate programs for students to enhance language and counseling skills in Spanish, thereby fostering language concordance in genetic counseling settings. With a response rate of 44.8% (26/58) across genetic counseling graduate programs, our study provides an overview of educational offerings in Spanish, encompassing patient‐facing, non‐patient‐facing, and combined opportunities. Of the programs that completed the survey, 73.1% (19/26) offer Spanish language opportunities. Several perceived benefits were identified by those that offer opportunities, including fostering cultural humility and diversity within the field, increasing awareness and accessibility of genetic counseling services, and facilitating involvement in research within minority groups. The information gathered from this study can be a resource for graduate programs seeking insights into effective strategies to incorporate Spanish language opportunities. Additionally, these results may also serve as a source of inspiration for students who want to apply their Spanish language skills in their training and future careers. Lastly, we propose ideas to enhance and expand the training of bilingual genetic counseling students in Spanish.

**What is known about this topic**

Presently, a limited number of genetic counseling programs promote Spanish language opportunities, as evidenced by presentations hosted by the National Society of Genetic Counselors (NSGC) and the Spanish Developmental SIG, and program website offerings. However, previous research emphasizes the advantages of language concordance in genetic counseling services, thus suggesting the need to incorporate Spanish language experiences into training programs.

**What this paper adds to the topic**

This research marks the first attempt to compile an overview of available Spanish language opportunities within genetic counseling programs. By doing so, it aims to offer an understanding of the current landscape of offerings and highlight potential pathways for other genetic counseling programs to include or increase their curricular offerings.

## 1 BACKGROUND

According to the United States Census Bureau data from 2022, the Hispanic population constituted the largest racial or ethnic minority group in the United States, comprising 19.1% of the population, with over 42 million individuals speaking Spanish as their primary language. Among these individuals, approximately 16 million have Limited English Proficiency (LEP) (U.S. Census Bureau, 2022), a term that describes an individual whose primary language is not English and who has difficulties communicating effectively in English (U.S. Department of Health & Human Services, 2023).

Language barriers are known to contribute to healthcare disparities experienced by LEP patients. These disparities manifest in various ways, such as fewer patient‐provider interactions, fewer healthcare visits, and lower rates of preventative screening (Smith, 2009). However, language barriers are not the only obstacle LEP individuals face when accessing healthcare services. Other factors such as low health literacy, cultural practices that discourage questioning healthcare providers, limited community support services (Foiles Sifuentes et al., 2020), and access to health insurance (U.S. Department of Health & Human Services, 2023) further hinder their ability to access and receive appropriate healthcare services.

In genetic counseling, the Reciprocal‐Engagement Model (REM) emphasizes the importance of the genetic counselor‐patient relationship by blending teaching and counseling while recognizing the uniqueness of each patient (Hartmann et al., 2013). Recent studies suggest that using this model in a language concordance setting facilitates communication by providing space for open discussion and clarification of questions which improves outcomes as patients feel emotionally supported and informed about the limitations and benefits of genetic testing (Jimenez et al., 2022). Additionally, the National Society of Genetic Counselors (NSGC) Code of Ethics emphasizes consideration and respect for each patient's individuality to uphold and appreciate the counselor‐client relationship (National Society of Genetic Counselors, 1992).

Overall, there is consensus on the benefits of language concordance between providers and patients. Previous research underscores the positive impact of delivering patient care in the patient's native language on physician‐patient communication and relationships. Although investigations into the physician‐patient relationship have been explored more extensively, these findings may serve as an example for enhancing communication and relationships in genetic counseling and other health professions (Jimenez et al., 2022).

The 2024 Professional Status Survey (PSS) conducted by NSGC revealed that only 4% (*n* = 110) of respondents self‐reported fluency in Spanish. Previous research has shown that in areas with a larger Hispanic population, there are more Spanish‐speaking genetic counselors in practice. However, the number of genetic counselors is inadequate to meet the LEP patients' needs (Augusto et al., 2018). This data highlights the current disparity between the population of Spanish‐speaking individuals and the number of Spanish‐speaking genetic counselors. Moreover, according to the data presented at the 2022 NSGC Annual Conference, during the session titled “Se Habla Español: Spanish Language Proficiency Training within Genetic Counseling Graduate Programs and Beyond,” (de Leon, Bergner, et al., 2022) only 2 out of the 52 genetic counseling programs accredited by the Accreditation Council for Genetic Counseling (ACGC) in the United States explicitly offer Spanish training on their respective websites (de Leon, Bergner, et al., 2022).

In general, there is a lack of clarity regarding the availability of Spanish language opportunities within genetic counseling programs, and accessing information about these prospects can be challenging. Anecdotal reports suggest that working closely with program leadership may offer more opportunities to utilize Spanish language skills (Clark et al., 2022).

As genetic counselors, our goal is to serve our patients and provide the best care possible. By diversifying our field, we can better meet the needs of LEP patients. Additionally, by gathering information on Spanish language opportunities offered by previous programs, we can create a valuable resource for future initiatives.

## 2 METHODS

### 2.1 Section I: Instrumentation

The study instrument was a cross‐sectional survey. The survey was created by the research team, consisting of two members from genetic counseling program leadership, a clinical genetic counselor with proficiency in Spanish, and the primary author – a native Spanish speaker and CCHI (Certification Commission for Healthcare Interpreters) certified medical interpreter. The 25‐item skip‐logic survey included dichotomous questions that were employed to gather insights into why graduate programs might not offer certain types of opportunities, the variety and timing of the opportunities, the number and types of supervisors/mentors available, the requirements for student participation in these opportunities, and demographic information. Likert scales were utilized to assess the frequency of Spanish language opportunities offered and the perceived levels of improvement resulting from these opportunities. Multiple‐answer questions were used to obtain a comprehensive understanding of the Spanish language opportunities available, including their locations, modes of service delivery, specialties, and details regarding the evaluation of students and supervisors/mentors for their Spanish proficiency, and optional open‐ended questions where respondents could elaborate on their answers or provide further information.

Due to the use of skip logic in the survey, not all questions were completed by each respondent. The survey was reviewed by the University of Wisconsin Survey Center and piloted by members of the study team. In addition, two bilingual genetic counselors and one genetic counseling student reviewed the survey for clarity of wording and proper use of skip logic.

### 2.2 Section II: Participants

#### 2.2.1 Inclusion criteria

Any member of the genetic counseling leadership team, including faculty, supervisors, and instructors, who could speak on behalf of the Spanish language opportunities from any ACGC‐accredited genetic counseling training program in the United States, was eligible for participation. We specifically sought one response per training program to ensure equal representation. We encouraged all programs to complete the survey regardless of the availability of Spanish language opportunities at their respective program.

#### 2.2.2 Exclusion criteria

The genetic counseling program from which this study was initiated did not provide a response.

### 2.3 Section III: Procedures

This study received an exemption from the University of Wisconsin‐Madison Institutional Review Board (2023‐1301). The survey was distributed through the Genetic Counselor Educators Association (GCEA) Listserv, formerly known as the Association for Genetic Counseling Program Directors (AGCPD). The survey was distributed as an online anonymous survey using Qualtrics software (Qualtrics, Provo, UT) and was available from November 2023 to January 2024. All data was securely stored.

Participants were required to provide their consent for the survey to proceed, which encompassed the survey's purpose, potential risks, benefits, and criteria for participation. This information was self‐reported.

### 2.4 Section IV: Data analysis

Data was analyzed and descriptive statistics were generated using Microsoft Excel and Qualtrics Data and Analysis tools, including Stats IQ and Crosstabs IQ.

Skip logic was employed in the survey, resulting in some questions having less than a 100% response rate. Open‐text responses obtained from participants were not coded or statistically analyzed since participants were not obligated to submit a response. Instead, some open‐text responses were included within the results section to provide context for the quantitative data. Responses to dichotomous closed‐ended questions were presented as percentages, while some of the multiple choice and Likert scale responses were grouped to provide a clearer overview of the data.

### 2.5 Section V: Definition of terms

The survey respondents were provided with the following definitions:

- *ACGC supervisor criteria*: A person with at least 1 year of experience as a clinical genetic counselor or post‐graduate experience and certified.
- *Advanced Spanish language proficiency*: Ability to fully and consistently interview patients or fluency akin to that of a native speaker.
- *Immersive Spanish rotations*: Rotation in which genetic counseling students provide genetic counseling services in Spanish at a primarily Spanish‐speaking clinic or hospital.
- *Intermediate Spanish language proficiency*: Ability to typically interview patients in Spanish.
- *Non‐patient‐facing opportunities*: Experiences where students could practice and improve their Spanish skills in a setting devoid of direct patient interaction.
- *Patient‐facing opportunities*: Experiences where students participated in genetic counseling appointments conducted in Spanish.
- *Supervisor/Mentor*: Includes individuals who meet ACGC criteria (e.g., someone with at least 1 year of post‐graduate experience and certified), or those who do not meet the aforementioned criteria (e.g., someone who lacks at least 1 year of post‐graduate experience but is certified), as well as any other healthcare professional, such as physicians, physician assistants, nurse practitioners, registered nurses, social workers, medical interpreters, etc. (Accredited Council for Genetic Counseling, 2020) who supervised genetic counseling students during their rotations.

## 3 RESULTS

### 3.1 Section I: Program demographics

**FIGURE 1:**
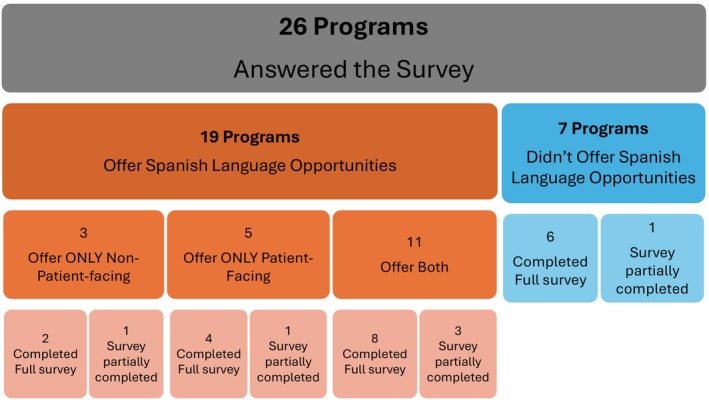
Survey response flowchart: completed vs. partially completed.

Data was obtained from 44.8% (26/58) of fully accredited genetic counseling programs in the United States. Among respondents, 76.9% (20/26) completed the entire survey, while 23.1% (6/26) did not. Although these three surveys lacked complete information, they were included in the analysis of this study (Figure 1).

The majority of respondents held positions as faculty or leadership within their respective genetic counseling program (95%, 19/20), with only one individual identifying as a genetic counselor supervisor or mentor (5%, 1/20). When asked how many genetic counselor supervisors/mentors were affiliated with the programs, 80% (16/20) reported having more than 30, and 20% (4/20) reported having less than 20.

### 3.2 Section II: Spanish language opportunities

Of the programs surveyed about Spanish language opportunities offered since the inception of their programs, 73.1% (19/26) of respondents reported having Spanish language opportunities.

We assessed the type of settings in which these opportunities were offered among the 19 programs. Around half of the programs (57.9%, 11/19) offered both non‐patient‐facing and patient‐facing opportunities, 26.3% (5/19) only had patient‐facing experiences, and 15.8% (3/19) only had non‐patient‐facing experiences.

#### 3.2.1 Non‐patient‐facing Spanish language opportunities (Figure 2)

**FIGURE 2:**
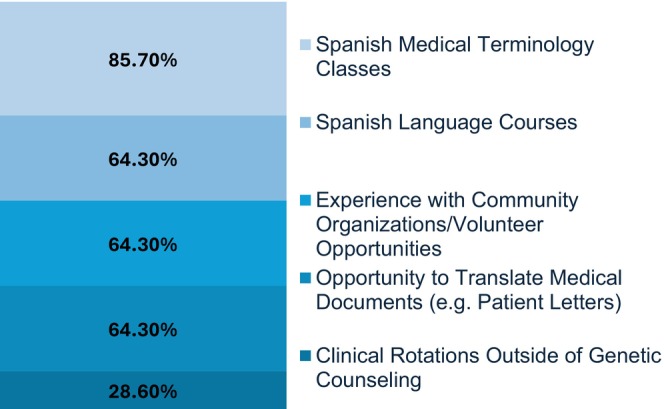
Non‐patient‐facing Spanish language opportunities.

Of the programs that reported Spanish language opportunities, many offered non‐patient‐facing experiences (*n* = 14). “Occasionally” or “frequently” offered opportunities included: Spanish medical terminology classes (85.7%, 12/14), Spanish language courses (64.3%, 9/14), experience with community organizations/volunteer opportunities (64.3%, 9/14), opportunities to translate medical documents (e.g., patient letters) (64.3%, 9/14), and clinical rotations outside of genetic counseling (28.6%, 4/14). Additionally, examples provided included facilitating testing for bilingual students to attain certifications (type not reported) and supporting a student's research project focused on the Hispanic community in their city, which involved conducting interviews in Spanish. More than half of the programs (71.4% 10/14) have been providing these opportunities for over 3 years, while 28.6% (4/14) of programs have offered such opportunities for 1–3 years.

#### 3.2.2 Patient‐facing Spanish language opportunities (Figure 3)

**FIGURE 3:**
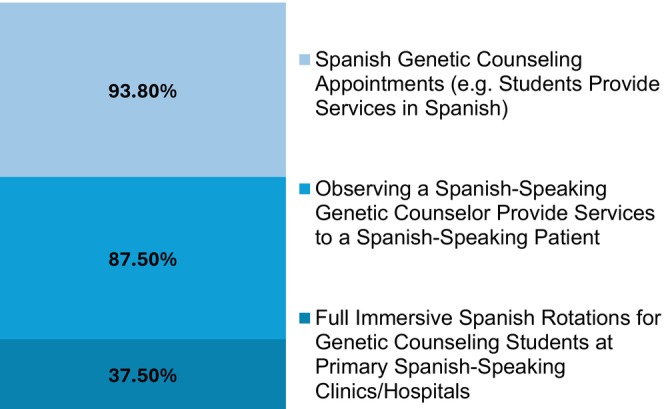
Patient‐facing Spanish language opportunities.

Of the programs that reported Spanish language opportunities, most offered patient‐facing opportunities (*n* = 16). The following were offered “occasionally” or “frequently”: Conducting Spanish genetic counseling appointments (e.g., students provide services in Spanish) (93.8%, 15/16), observing a Spanish‐speaking genetic counselor provide services to a Spanish‐speaking patient (87.5%, 14/16), and full immersive Spanish rotations for genetic counseling students at primarily Spanish‐speaking clinics/hospitals (37.5%, 6/16).

Two programs commented that they allow students to observe or have limited participation in appointments where the patient spoke Spanish, but the supervisor did not. Consequently, they completed the appointment with the assistance of a Spanish medical interpreter. One program shared:

[…]genetic counseling students provide genetic counseling services with the assistance of a Spanish interpreter regularly assigned to the clinic and also observe the Spanish‐speaking provider speaking to the Spanish‐speaking patient; depending on students' fluency in Spanish, [there may be] occasional opportunity to provide portions of genetic counseling session in Spanish under supervision of Spanish‐speaking GC or medical geneticist.

One program reported that their students may seek summer rotations in a Spanish‐speaking country. However, students are responsible for arranging their placement.

Most programs reported providing these opportunities for three or more years (75%, 12/16), while 25% (4/16) have offered them for 1–3 years. Additionally, when we asked when these opportunities are being offered to students (*n* = 14), 92.9% (13/14) offered these opportunities by the second year of training. Over half (64.3%, 9/14) offered Spanish language opportunities during the 1st year of training.

The service delivery modes utilized during these patient‐facing opportunities were consistent between all programs. All programs that utilized academic hospitals (*n* = 15) offered in‐person opportunities (15/15), 73.3% (11/15) via telehealth (video and audio), and 60% (9/15) via telephone only. Those who utilized Public Hospitals (including federally qualified health centers such as community health centers, *n* = 9) provided services in‐person (9/9), 77.8% (7/9) via telehealth, and 66.7% (6/9) via telephone only. Those who utilized Private Hospitals (*n* = 6) all offered services in‐person (6/6), 66.7% (4/6) via telehealth (video and audio), and 66.7% (4/6) via telephone only. None of the programs reported utilizing laboratories or telehealth/consulting/utilization management settings for these experiences.

Genetic counseling specialties utilized for patient‐facing opportunities included (*n* = 15): general genetics (adult and pediatric) (86.7%,13/15), prenatal (80%, 12/15), oncology (53.3%, 8/15), neurogenetics (20%, 3/15), Newborn screening (20%, 3/15), laboratory (13.3%, 2/15), metabolic/biochemical genetics (13.3%, 2/15), cardiology genetics (6.7%, 1/15), personalized/ precision medicine (6.7%, 1/15), hematology (6.7%, 1/15), ophthalmology (6.7%, 1/15), fertility clinics (6.7%, 1/15), psychiatric (6.7%, 1/15), and other specialties (6.7%, 1/15).

We surveyed programs that had never offered Spanish language opportunities (*n* = 7) about barriers that prevented them from doing so. Barriers included limited resources and funding (71.4%, 5/7), time constraints within their curriculum (71.4%, 5/7), and lack of qualified Spanish‐speaking supervisors (57.1%, 4/7). None of the programs reported a lack of demand for Spanish language opportunities. When analyzing the relationship between the barriers preventing programs from offering Spanish language opportunities and the total number of genetic counselor supervisors/mentors reported by each program, we observed that the responses were evenly distributed between programs with less than ten supervisors/mentors and those with over 40 supervisors/mentors.

### 3.3 Section III: Supervisors/mentors of Spanish language opportunities

Individuals providing supervision/mentorship in patient‐facing Spanish language opportunities (*n* = 14) varied in experience. All programs reported using genetic counselors who meet the ACGC criteria for genetic counselor supervisors. However, approximately half of the programs (53.3%, 8/14) utilize other healthcare professionals, such as physicians, physician assistants, and nurses. Two programs reported using genetic counselors who do not meet ACGC criteria (14.3%).

Most programs (78.6%, 11/14) reported having 1–5 Spanish proficient supervisors/mentors. Only two programs reported having 6–10 Spanish proficient supervisors/mentors (14.3%, 2/14), and one program (7.1%, 1/14) had no access to Spanish proficient supervisors/mentors.

When asked about the methods used to evaluate the Spanish proficiency of supervisors/mentors, respondents indicated that 35.7% (5/14) of programs required their Spanish‐speaking supervisors/mentors to be certified medical interpreters, and 5/14 programs did not assess Spanish proficiency.

When we asked about the language used to provide feedback to the students, most programs reported feedback in English (85.7%, 12/14), whereas 28.6% (4/14) also reported allowing it in the student's preferred language.

For those programs that used English‐speaking‐only supervisors in patient‐facing Spanish language opportunities (*n* = 12), respondents reported that the majority offered these opportunities with the assistance of a certified medical interpreter (84.6%, 11/12), and two programs also used the assistance of bilingual staff (15.4%).

### 3.4 Section IV: Students participating in Spanish language opportunities

**FIGURE 4:**
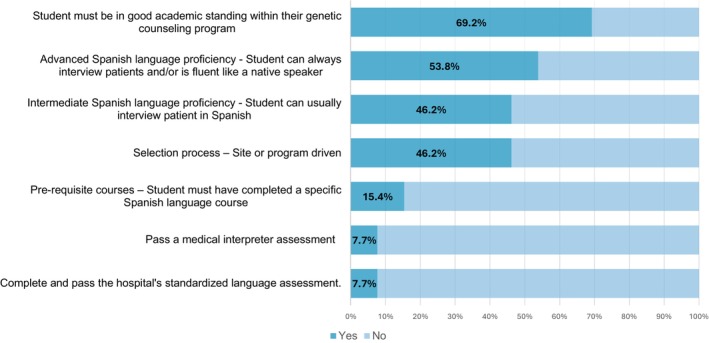
Students requirements to participate in patient‐facing Spanish language opportunities.

When we inquired about the requirements for students to participate in Spanish language opportunities, programs (*n* = 13) (Figure 4) reported a variety of requirements including students maintaining good academic standing (69.2%, 9/13), advanced Spanish language proficiency (53.8%, 7/13), intermediate Spanish language proficiency (46.2%, 6/13), selection process driven either by the site where the rotation took place or by the program itself (46.2%, 6/13), prerequisite courses such as a specific Spanish language course (15.4%, 2/13), successful completion of a medical interpreter assessment (7.7%, 1/13), and successful completion of the hospital's standardized language assessment (7.7%, 1/13).

Programs evaluated a student's Spanish language proficiency in various ways. 38.5% (5/13) of the programs required students to meet one or more of the following criteria: Pass a standardized language test, complete an oral proficiency interview, report any previous experience as a Spanish interpreter (not certified), possess an undergraduate or graduate degree in Spanish, or hold certification as a medical interpreter. The remainder (61.5%, 8/13) did not require a proficiency test. We also asked if students had any requirements to complete during these patient‐facing Spanish language opportunities, such as providing genetic counseling services, counseling outlines, or creating resources or visual aids in Spanish. Many (61.5%, 8/13) reported no required tasks for the students. However, one program specified that although there are no specific requirements, students are encouraged to get involved as much as they feel comfortable. Several (38.5%, 5/13) required students to provide genetic counseling services in Spanish. Of these five programs, one reported requiring students to create resources/visual aids in Spanish and translate patient letters into Spanish. Another program also reported requiring outlines in Spanish.

### 3.5 Section V: Programs reflection on offerings of Spanish language opportunities

We asked programs (*n* = 16) if they would change anything about their current Spanish language opportunities. Most programs expressed interest in incorporating changes (68.8%, 11/16). The suggested changes included providing more Spanish language opportunities, increasing the number of Spanish‐speaking supervisors, exploring Spanish language opportunities in different regions, prioritizing language concordance in the hiring process of healthcare institutions, increasing funding and time to support these opportunities, offering Spanish language opportunities more frequently, and addressing the lack of accessibility to medical Spanish classes.

**FIGURE 5:**
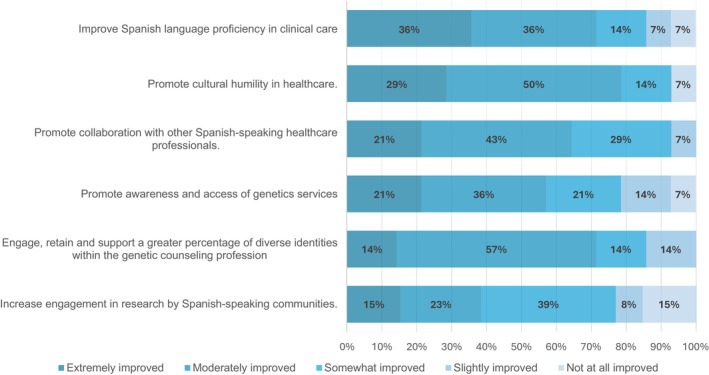
Degree to which participants felt Spanish language opportunities improved the field of genetic counseling.

Of the 14 programs that provided more information about their impressions on benefits and limitations these experiences could offer, most programs noted “moderate” to “extreme” benefits (Figure 5) in the following: promoting cultural humility in healthcare (78.6%, 11/14), helping engage, retain and support more diverse identities within the genetic counseling profession (71.4%, 10/14), improving Spanish language proficiency in clinical care (71.4%, 10/14), promoting collaboration with other Spanish‐speaking healthcare professionals (64.3%, 9/14), promoting awareness and access of genetic services (57.1%, 8/14), and increasing engagement in research by Spanish‐speaking communities (35.7%, 5/14). However, a few programs (14.3%, 2/14) reported “no” to “slight” improvement in those areas. We sought to ascertain whether the types of Spanish language opportunities provided (patient‐facing, non‐patient‐facing, or both) by programs influenced the perceptions of the benefits or limitations they observed. Among the programs offering patient‐facing only and both patient‐facing and non‐patient‐facing opportunities (*n* = 10), the majority (90%, 9/10) reported observing “moderate” to “extreme” improvements in language proficiency, cultural humility, increasing engagement in research, promoting awareness, and access to genetic services, as well as strengthened support for diversity and inclusion within the field of genetic counseling. In contrast, the program (10%, 1/10) that exclusively offers non‐patient‐facing opportunities noticed “slight” to “no” improvement in those same areas.

Lastly, we asked responders to share any other thoughts they have related to Spanish language opportunities in genetic counseling training programs. Examples included:

I wish we had more time to offer additional resources and opportunities to our students. For the students who are bilingual, we also run into some legality issues into when and to what degree they are allowed to use Spanish – we are hoping that by getting them certified it will help bypass these issues in rotation sites – but that doesn't negate the fact that most supervisors don't speak Spanish. In general[,] we are focusing on providing our bilingual students as many opportunities as we can (i.e., being given priority in obtaining Spanish‐speaking supervisors and access to Hispanic patient populations), and focusing on community outreach initiatives where all students can interact with our city's Hispanic communities (and allowing our bilingual students to use Spanish in these situations).

Another program provided other creative ways to improve their Spanish language opportunities:

Provide students with opportunities to engage with patients as they are able. Create simulated cases and hire an SP [standardized patient] to present the case in Spanish, [.] [T]here is evidence based research which shows that simulation also allows a provider to have more awareness of their own language ability accordingly, which is also important for patient care.

One program mentioned:

There is probably more interest in Spanish‐language opportunities than ability to offer them.

## 4 DISCUSSION

Our study group included nearly half of the accredited genetic counseling programs in the United States and aims to provide insight into the current state of Spanish language opportunities within genetic counseling programs.

### 4.1 Landscape of Spanish language opportunities

Overall, our study found that many genetic counseling graduate programs offer Spanish language opportunities, demonstrating innovative strategies despite challenges like the known shortage of Spanish‐speaking supervisors. While there are discrepancies in language proficiency assessment for supervisors/mentors and students, these opportunities still yield numerous benefits.

Both a prior online session hosted by the NSGC Spanish Developmental SIG (de Leon, Bergner, et al., 2022) and a review of publicly available program information noted that only a couple of genetic counseling training programs openly advertise offering Spanish language opportunities. Recent research has shown that genetic counseling graduate programs offer support to bilingual students during training (Franca et al., 2024); however, this encompasses less than half of the graduate programs, as per our data. Additionally, our study revealed that many programs in the United States have successfully implemented innovative approaches despite reported challenges, such as the lack of Spanish‐speaking supervisors/mentors and limitations in resources, funding, and time, demonstrating that it is feasible to offer these opportunities.

Our study also aligns with existing evidence indicating a scarcity of Spanish‐speaking supervisors in genetic counseling training programs (Augusto et al., 2018; NSGC PSS, 2024). Consequently, nearly half of the programs surveyed that provide these opportunities rely on other healthcare professionals to facilitate these experiences, including utilizing medical interpreters when no Spanish‐speaking supervisors/mentors are available. Reliance on other healthcare professionals provides an example of the creative solutions used to address these challenges. One program explored utilizing international resources to accommodate these Spanish language opportunities. However, this approach may not be feasible for all students due to associated costs and travel concerns. A more accessible strategy could involve collaboration between graduate programs or forming partnerships with Spanish‐speaking genetic counselors to offer supervision in regions lacking Spanish‐speaking genetic counselors in a clinical setting or utilizing Spanish‐speaking standardized patients to enrich the learning experiences of bilingual students.

Furthermore, our investigation unveiled a discrepancy in how programs evaluate Spanish proficiency among supervisors/mentors, with half imposing requirements such as medical interpreter certification or standardized language testing, while the remaining do not mandate any verification. A similar inconsistency was noted in assessing students' language proficiency for participation in these opportunities. This variation could stem from the diversity within institutional and state regulations. While a one‐size‐fits‐all solution may not exist, perhaps developing a roadmap to navigating legal regulations could enhance the availability of Spanish language opportunities across genetic counseling graduate programs. Such roadmaps can alleviate the workload for programs seeking to integrate these opportunities and ensure consistency across programs. This clarity of expectations benefits supervisors/mentors and students while also ensuring that limited English proficiency (LEP) patients receive equitable services alongside all patients.

As anticipated, our data affirmed the benefits of Spanish language opportunities (Jimenez et al., 2022), especially when patient‐facing options are available. These experiences play a vital role in enhancing the accessibility and diversity of the genetics field. For students, they offer a chance to hone their genetic counseling skills in Spanish, better preparing them for clinical practice (National Society of Genetic Counselors & Hernandez, 2018) by fostering cultural humility and diversity within the field to enhance awareness and access to genetic counseling services, as well as facilitating involvement in research within minority groups. Similarly, patients benefit by gaining access to genetic counseling services in their preferred language, facilitating effective communication, and fostering a feeling of comfort and trust. Previous research on language concordance in genetic counseling services (de Leon, McCarthy Veach, et al., 2022; Jimenez et al., 2022) highlights the reliance of genetic counselors on the REM to allow for effective communication and rapport building with patients.

Our study supports that more Spanish language opportunities exist than advertised, challenging the perception of limited availability and prompting a reconsideration of how these programs communicate and promote their language offerings. Many genetic counseling programs have successfully navigated the challenges encountered, paving the path for other institutions to support bilingual students and prepare them to provide genetic counseling services in languages spoken by their patients. Providing these opportunities enhances access to care and can significantly improve emotional and personal support for patients (Jimenez et al., 2022), ultimately enhancing their overall experience.

By promoting these Spanish language opportunities, we not only contribute to making the field of genetic counseling more accessible and diverse but also highlight the advocacy role within genetic counseling. Genetic counselors strive to recognize and respect each person's individuality (NSGC – Code of Ethics), and offering services in their preferred language is integral to respecting a person's individuality emphasizing the importance of cultural competency and sensitivity in providing genetic counseling services, and ultimately fostering a more inclusive and patient‐centered approach to healthcare.

### 4.2 Study limitations

Since we did not collect data from all the ACGC‐accredited programs in the United States, it is important to acknowledge that our findings may have limitations, potentially overlooking additional offerings or innovative ideas.

It is also important to note that most of the information we collected reflects the perspective of genetic counseling program faculty or leadership members who responded to the survey. This limited perspective means we may have missed insights from others who work closely with students and patients in these programs. Additionally, we did not collect information directly from students or patients.

Furthermore, we did not inquire about the age of the programs. Therefore, younger graduate programs may not have had the opportunity to explore these types of Spanish language experiences for their students.

Despite these limitations, the collected data offers valuable insights for future research and provides practical ideas for genetic counseling programs interested in integrating Spanish language opportunities for their trainees and bilingual students seeking to apply their language skills.

### 4.3 Future directions

As we explore potential avenues for future research and initiatives based on the feedback received through this survey, several ideas emerge, including: Collecting data from the remaining accredited programs, surveying students who participated in these opportunities to get a sense of perceived benefits during training and as practicing genetic counselors, patient input on benefits and limitations to these experiences, and exploring the development of tools to enhance the accessibility of these opportunities.

Another aspect worth considering is the introduction of a genetic counseling terminology course, as proposed in the survey and supported by prior research and the NSGC on‐demand video “¿Que Dijo? Identifying Resources to Support Spanish Speaking Patients within Genetic Counseling” (Clark et al., 2022; Franca et al., 2024). Further investigation is necessary to determine interest and evaluate the feasibility of implementing such a course. One potential approach could be to offer the course to all bilingual students during their genetic counseling training or as a postgraduate continuing education option. This could foster collaboration between programs and enhance accessibility, particularly for programs with limited resources or few Spanish‐speaking students.

Further research is also needed to explore how to expand the number of bilingual genetic counseling supervisors. Numerous studies have shown that BIPOC (Black, Indigenous, and People of Color) students at high schools and colleges are less likely to be aware of genetic counseling compared to their white peers (Gerard et al., 2018; Oh & Lewis, 2005). Additionally, individuals from racial and ethnic minorities tend to become acquainted with genetic counseling later in life (Alvarado‐Wing et al., 2021). These barriers may contribute to this disparity. Valuable lessons can be learned from successful initiatives targeting racial and ethnic diversity in fields such as nursing and medicine. These initiatives typically encompass educational interventions that prepare students to understand the curriculum with mentorship programs during high school (Karen Devereaux et al., 2013; Patel et al., 2015). Some of these resources are available to prospective genetic counseling students through organizations such as the “Minority Genetic Professional Network.” Despite these resources, more effort is needed to implement early exposure to genetic counseling and expand mentoring programs. Another critical aspect is retaining Spanish‐speaking genetic counselors, particularly as the field experiences continued growth and diversification. Despite the lack of research on this topic, exploring retention strategies for Spanish‐speaking genetic counselors could be a valuable area for future investigation.

Lastly, limited research has been conducted on Spanish or other language opportunities during genetic counseling training, indicating the need for further investigation and a better understanding of them.

### 4.4 Practice implication

Our study's exploration of Spanish language opportunities yields significant implications for genetic counseling programs and students. For programs, it provides invaluable insights into the landscape of available offerings, supporting the integration of such opportunities into their curricula. Moreover, it may serve as a beneficial resource for students navigating communication with their respective programs.

Through collaborative learning and shared expertise, genetic counseling programs can enhance their current offerings for Spanish language opportunities and work collectively to better understand legal requirements or institutional policies. This collaborative endeavor will allow students to receive enriching educational experiences and patients to receive consistent, high‐quality services across various programs. By fostering a culture of shared knowledge and best practices, the genetic counseling community can effectively serve diverse populations and promote equitable access to care.

## 5 CONCLUSION

Our study provided insight into the current offerings for bilingual genetic counseling students aspiring to apply their language skills in practice. While only two genetic counseling training programs openly advertised Spanish language opportunities, our research revealed that more programs offer these opportunities, and various experiences are available to students at many graduate programs.

Lastly, although this research study focuses on Spanish language opportunities, we hope it applies to opening opportunities for other languages.

## AUTHOR CONTRIBUTIONS

Maria Katia Vine contributed to the conception, study design, data collection, data analysis and interpretation, and drafting of the manuscript. Laura Birkeland, Ashley Kuhl, and Elizabeth Kellom, contributed to the conception, study design, data analysis and interpretation, and manuscript revision. Laurie Simone contributed in the tools and data analysis, data interpretation, and manuscript revision. Charité N. Ricker contributed to data analysis and interpretation, and manuscript revision. All authors confirm they had full access to all the data in the study and accept responsibility for its integrity. Furthermore, all authors gave final approval for this version to be published and agree to be accountable for all aspects of the work, ensuring that any questions regarding the accuracy or integrity of any part of the work are properly investigated and resolved.

## CONFLICT OF INTEREST STATEMENT

Maria K Vine, Laura Birkeland, Elizabeth Kellom, Ashley Kuhl, Laurie Simone, and Charité Ricker had no conflicts of interest.

## ETHICS STATEMENT

Human studies and informed consent: This study was approved by and conducted in accordance with the ethical standards of the University of Wisconsin Institutional Review Board (IRB #2023‐1301). Informed consent was obtained from all participants prior to their completion of the survey.

Animal studies: No non‐human animal studies were carried out by the authors for this article.

## Data Availability

The data supporting the findings of this study are available upon request from the corresponding author [MV].
